# Chemical Elements and the Quality of Mānuka (*Leptospermum scoparium*) Honey

**DOI:** 10.3390/foods10071670

**Published:** 2021-07-20

**Authors:** Alexandra Meister, Maria Jesus Gutierrez-Gines, Aydin Maxfield, Sally Gaw, Nicholas Dickinson, Jacqui Horswell, Brett Robinson

**Affiliations:** 1 School of Physical and Chemical Sciences, University of Canterbury, Christchurch 8041, New Zealand; sally.gaw@canterbury.ac.nz (S.G.); brett.robinson@canterbury.ac.nz (B.R.); 2 Institute of Environmental Science and Research Ltd. (ESR), Christchurch 8041, New Zealand; Maria.Gutierrez-Gines@esr.cri.nz; 3 Lowe Environmental Impact Ltd., Palmerston North 4410, New Zealand; aydin@lei.co.nz; 4 Department of Ecology, Lincoln University, Lincoln 7647, New Zealand; Nicholas.Dickinson@lincoln.ac.nz; 5 Ministry for Primary Industries, Wellington 6140, New Zealand; Jacqui.Horswell@mpi.govt.nz

**Keywords:** dihydroxyacetone, methylglyoxal, non-peroxide antimicrobial activity, mānuka honey, New Zealand

## Abstract

Soil properties in the foraging range of honeybees influence honey composition. We aimed to determine relationships between the antimicrobial properties of New Zealand mānuka (*Leptospermum scoparium*) honey and elemental concentrations in the honey, plants, and soils. We analyzed soils, plants, and fresh mānuka honey samples from the Wairarapa region of New Zealand for the chemical elements and the antimicrobial activity of the honey as indicated by methylglyoxal (MGO) and dihydroxyacetone (DHA). There were significant negative correlations between honey MGO and the concentrations of Mn, Cu, Mg, S, Na, Ba, K, Zn, and Al. These elements may provide a low-cost means of assessing mānuka honey quality. For individual elements, except for K, there were no correlations between the honeys, plants, and soils. Soil nitrate concentrations were negatively correlated with concentrations of MGO and DHA in the honey, which implies that soil fertility may be a determiner of mānuka honey quality.

## 1. Introduction

*Leptospermum scoparium* J.R. et G. Forst. is the most widespread indigenous shrub species in New Zealand and is commonly known as mānuka or tea tree [1]. It is a member of the Myrtaceae family and one of 13 species in the *Leptospermum myrtifolium* subgroup [2]. Economically, *L. scoparium* is important due to production of its essential oil and mānuka honey. Most of the 8065 tons of honey exported from New Zealand in 2019, which created a revenue of NZD 355 M (approximately USD 250 M), was mono- or multi-floral mānuka honey [3].

Honey is naturally antiseptic because it is osmotically unfavorable to microbial growth and has a low pH [4]. Whilst honeys typically contain the antimicrobial compound hydrogen peroxide (H_2_O_2_), mānuka honey is unusual due to its non-peroxide antimicrobial activity (NPA) [5]. The dominant component responsible for NPA in mānuka honey is methylglyoxal (MGO) [6]. Other compounds, including leptosin and various phenolics, synergistically modulate mānuka honey NPA [7]. MGO is formed in the honey due to non-enzymatic dehydration of dihydroxyacetone (DHA) from *L. scoparium* nectar [8,9]. Therefore, the concentration of MGO increases simultaneously with a decrease in DHA during maturation and storage of mānuka honey in warm temperatures [10]. Mānuka honey can inhibit a range of pathogenic bacteria genera, including *Enterococcus*, *Pseudomonas*, *Staphylococcus*, and *Streptococcus*, among others [7]. Antimicrobial action may occur due to the disruption of regular cell division, impairment of cellular integrity, and reduction in cellular motility [11]. Its distinct antimicrobial characteristics mean that the market value of mānuka honey is primarily determined by its NPA, which is often commercially expressed as Unique Mānuka Factor (UMF™), though other marketing terms exist [6]. DHA concentration in *L. scoparium* nectar is affected by a plethora of genetic and environmental factors [12]. Although these have yet to be quantified, they may include the concentrations of the chemical elements in the nectar.

Soil is the ultimate source of many elements in the floral nectar [13,14]. The concentration of elements in honey is affected by soil characteristics, and honey composition can be used for geographical discrimination or as a soil element indicator [13,15,16,17]. The response of *L. scoparium* to soil properties is cultivar-dependent [18]. However, Williams et al. [19] found that soil properties do not affect the concentration of DHA in *L. scoparium* nectar. This is consistent with other studies which show that genetic factors and provenances are more relevant for *L. scoparium* nectar DHA [20,21]. Noe et al. [22], however, reported that *L. scoparium* nectar DHA varies more among plants than among sites. It is unclear how the environment affects the composition of *L. scoparium* nectar and, subsequently, mānuka honey.

There are no reports of the effect of soil and plant elemental concentrations on the elemental composition and NPA of mānuka honey. *L. scoparium* is typically found growing on low fertility soils [23], and increased soil fertility accelerates growth of the plant [24]. Nickless et al. [18] showed that increased soil nutrient concentration also improved floral density of *L. scoparium*. The link appears to be missing between soil parameters and mānuka honey MGO. Although DHA contents of *L. scoparium* plants within 1000 m from the apiary correlate well with MGO in honey [25], actual honey MGO contents are typically lower than nectar-DHA-based estimates [26]. Mānuka honey is rarely collected from 100% *L. scoparium* nectar, so it is vital for beekeepers to increase the availability of DHA-containing nectar to honeybees in order to achieve high MGO mānuka honeys [26]. Higher concentrations of soil nutrients within the foraging range of honeybees might therefore result in increased availability of *L. scoparium* nectar by increasing the floral density. 

We aimed to determine the effect, if any, of the elemental composition of soils, plants, and honey on the quality of mānuka honey as indicated by MGO and DHA. Additionally, we sought to compare the chemical composition of mānuka honey from different sites in the Wairarapa region of New Zealand.

## 2. Materials and Methods

### 2.1. Sample Collection

**Figure 1:**
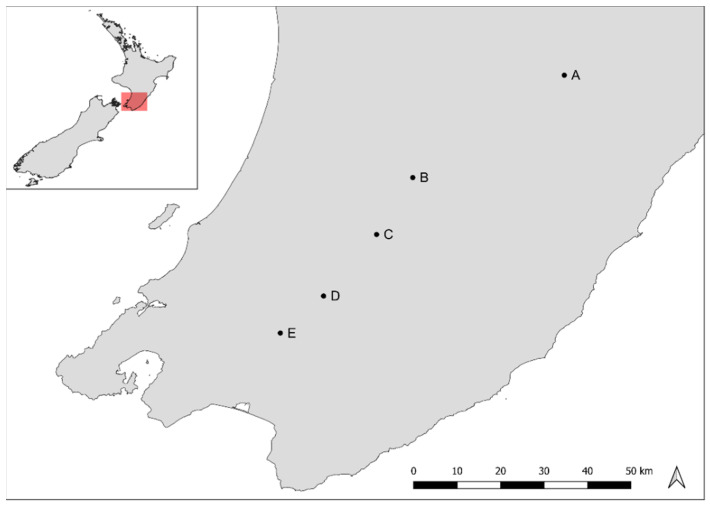
Sampling sites (A–E) in the Wairarapa region of New Zealand.

Soil, plant foliage, and honey samples were collected from five sites in the Wairarapa region in the lower North Island of New Zealand (Figure 1). Soil and foliage samples were collected in April 2014. Five *L. scoparium* plants were sampled per hive location. Plants were between 1.5 and 3 m tall. Foliage was sampled at 2 m above ground where possible. A representative sample was taken by combining 10 individual twigs per tree. A soil sample was taken at the base of each sampled plant, within 0.5 m from the base. All sampling sites were within 1 km from the hive. This is within the foraging range of the honeybee *Apis mellifera* [27]. Soil and plant samples were immediately sent to the laboratory for further processing. Soils were kept cold in insulated containers with ice packs. Raw honey samples were extracted by Watson & Son Ltd. (Masterton, New Zealand, now Oha Honey LP) in January–February 2014. Honey samples from locations B, C, and D are composite samples, as multiple hives were within 1 km from each other at these sites.

### 2.2. Honey Analysis

Honey samples (0.5 g) were digested in 8 mL ARISTAR^®^ 69% HNO_3_ with a microwave (MARS Xpress, CEM Corporation, Matthews, NC, USA). Total concentrations of Al, As, B, Ba, Ca, Cd, Cr, Cu, Fe, K, Mg, Mn, Na, P, Pb, S, and Zn were determined by ICP-OES (Varian 720-ES, Agilent Technologies, Santa Clara, CA, USA). Concentrations of honey DHA and MGO were simultaneously determined by HPLC at Watson & Son Ltd. (Masterton, New Zealand, now Oha Honey LP) following the method described by Windsor et al. [28]. The HPLC system consisted of a Dionex ACC-3000 autosampler, a Dionex LPG-3400SD quaternary pump, and a Dionex VWD-3100 detector (λ = 263 nm) (Thermo Fisher Scientific, Waltham, MA, USA). A Phenomenex Synergi Fusion 75 mm × 4.6 mm, 4 µm, 80Å reversed-phase column was used with a Phenomenex Synergi 4 mm × 3 mm guard column (Phenomenex, Torrance, CA, USA).

### 2.3. Soil and Plant Analysis

NH_4_^+^ and NO_3_^−^ were extracted from fresh soil with 2 M KCl and analyzed by a flow injection analyzer (FIAstar 5000, FOSS, Hillerød, Denmark) [29]. Soils were dried at room temperature until a constant weight was achieved and then were sieved to 2 mm. Soil pH was determined in a 1:2.5 water extract [30]. In all, 0.5 g of soil was digested by microwave (CEM MARS Xpress) in 5 mL ARISTAR^®^ 69% HNO_3_ and 1 mL ARISTAR^®^ 30% H_2_O_2_. Pseudo-total Al, As, B, Ca, Cd, Cr, Cu, Fe, K, Li, Mg, Mn, Na, Ni, P, Pb, S, Sr, and Zn concentrations in the digest were analyzed by ICP-OES (Varian 720-ES). The exchangeable element fraction was determined in a 0.05 M Ca(NO_3_)_2_ extract using ICP-OES (Varian 720-ES) [31]. Total C and N were determined using an Elementar Vario Max CN elemental analyzer (Elementar, Langenselbold, Germany).

Foliage samples were rinsed with deionized water and dried at 65 °C until a constant weight was achieved. Leaves were separated from the twigs and ground using a Retch ZM200 mill. In all, 0.5 g of ground foliage was digested in 8 mL ARISTAR^®^ 69% HNO_3_ by microwave (CEM MARS Xpress). Total Al, As, B, Ca, Cd, Co, Cr, Cu, Fe, K, Li, Mg, Mn, Na, Ni, P, Pb, S, Sr, and Zn concentrations in the digest were determined by ICP-OES (Varian 720-ES) [31]. Total C and N concentrations were determined using an Elementar Vario Max CN elemental analyzer.

### 2.4. Quality Control

Wageningen Evaluating Programmes for Analytical Laboratories (WEPAL) certified reference materials ISE 921 and IPE 100 were used for quality assurance in soil and plant digestions. Recoveries ranged from 91% to 108% of certified values. Analytical blanks were included in all analyses.

### 2.5. Statistical Analysis

Data were analyzed using R version 4.0.5. [32]. One-way analysis of variance (ANOVA) followed by Tukey’s honestly significant difference (HSD) test was used to assess site differences using the package multcomp. Data were log-transformed where the assumption of normality was not met. The significance level for all statistical analyses was *p* ≤ 0.05. A principal component analysis (PCA) was carried out for honey variables using the package factoextra. The packages ggplot2 and ggpubr were used to visualize results of correlation analysis. No statistical analyses were performed on honey Cd, As, and Pb concentrations as these were below detection limits (<0.001 mg kg^−1^).

## 3. Results and Discussion

### 3.1. Soil and Plants

The soils were typically yellow-brown loams [33]. Soil pH at all sites was moderately to strongly acidic (Table S1) and lower than typical soil pH under New Zealand pasture, which ranges from 4.8 to 6.9 [31]. *L. scoparium* is commonly found growing on acidic soils [34]. At low soil pH, some trace element cations, including Al, Cu, Fe, Mn, and Zn, are more soluble and more plant-available [35]. We found soil pH was negatively correlated with some extractable elements (Table S2), which included Al (*r* = −0.71, *p* ≤ 0.001), Cd (*r* = −0.49, *p* ≤ 0.005), Cr (*r* = −0.51, *p* ≤ 0.001), and Zn (*r* = −0.45, *p* ≤ 0.005). Soils were generally low in P (452–878 mg kg^−1^) when compared with New Zealand pasture soil, but had similar concentrations of N and C [31].

Plant samples were particularly high in Mn and Ni (Table S3) when compared with average plant shoots [36]. In contrast, the concentration of some of the P, K, and Mg was lower than average for plant dry matter concentrations [36]. Most plant parameters did not differ significantly between sites (C, N, C/N, As, B, Ca, Cd, Cu, Ni, and Zn). Site C differed from all other sites as it had significantly higher K and significantly lower Mn foliage concentrations.

Plant Mn in this study ranged from 53 to 1309 mg kg^−1^ with a median of 194 mg kg^−1^. This is comparable to studies by Gutierrez-Gines et al. [37] and Reis et al. [38], who reported 185–292 and 186–331 mg Mn kg^−1^ in non-fertilized *L. scoparium*, respectively. Both of these studies measured increased leaf Mn concentrations following the application of biosolids, which potentially reach phytotoxic levels at >400 mg Mn kg^−1^ [39]. In the present study, sites A and E exceeded this threshold with an average Mn concentration of 874 and 448 mg kg^−1^, respectively.

For the major nutrients N and P, higher soil concentrations that increase plant growth may result in a dilution of other elements in plant tissues [40]. However, we found significant positive correlations between extractable soil P and plant P (*r* = 0.42, *p* ≤ 0.005), extractable soil Mg and plant Mg (*r* = 0.40, *p* ≤ 0.01), and extractable soil Mn and plant Mn (*r* = 0.34, *p* ≤ 0.05).

### 3.2. Honey

Table 1 reports honey MGO, DHA, and elemental concentrations. The DHA concentrations in this study were similar to those reported in fresh mānuka honey by Atrott et al. [10] and Adams et al. [8]. MGO concentrations were in the low range, lower than those in fresh mānuka honeys (309–658 mg kg^−1^) reported by Stephens et al. [41]. There was a strong positive correlation (*r* = 0.99, *p* ≤ 0.001) between DHA and MGO, with MGO concentrations being on average 7% of DHA concentrations. Therefore, we henceforth report MGO as an indicator of mānuka honey quality.

The elemental concentrations in the honey were comparable to analyses of other fresh mānuka honey in New Zealand [42]. However, Na and Al were four and seven times higher, respectively, than concentrations reported by Vanhanen et al. [42]. The elemental concentrations in our fresh honey samples were lower than those in commercially available New Zealand mānuka honeys reported by international studies [43,44,45,46]. The concentration of chemical elements in honey may increase following moisture reduction during honey processing [47].

The most abundant element in the tested honeys was K, followed by Ca and P. These were the overall most abundant elements in mono-floral New Zealand honeys analyzed by Vanhanen et al. [42]. They were also the most prominent elements in various international honey types [48]. Mānuka honey in this study had higher concentrations of elements, particularly Na, Ca, Mg, P, and Mn, compared to other honey types [49]. In the case of Mn, mānuka honey was shown to have higher concentrations than other New Zealand mono-floral honeys, with the exception of rewarewa honey [42].

An increased elemental concentration as such can be beneficial for human nutrition [50], although, given the average daily consumption of honey (0.1–0.8 kg per annum), human health benefits from the elements contained in honey are negligible [49,51]. Heavy metals such as, Cd, and Pb were below the detection limit (<0.001 mg kg^−1^) in this study and therefore not of significance to human health.

**Figure 2:**
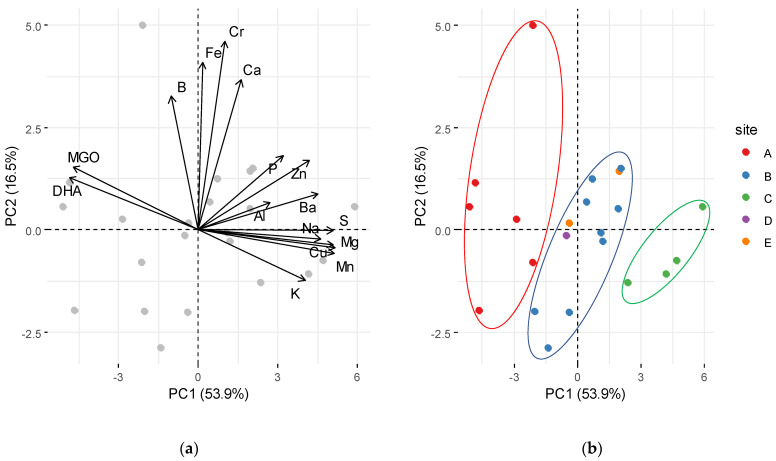
Principal component analysis (PCA) describing the variation of honey MGO, DHA, and elemental concentrations: (**a**) loading plot; (**b**) score plot. The ellipses are eye-guides to delineate the three distinct groupings.

A PCA was used to investigate similarities between mānuka honey quality parameters and elemental composition between sites (Figure 2). The honeys can be separated into three distinct groups at sites A, B, and C, with honey DHA and MGO having a positive weighting in PC1 (explaining 53.9% of variance) and other elements, dominated by Mg, Mn, Cu, Ba, Na, Zn, and K, having negative weightings. PC2 (explaining 16.5% of variance) separated the sites mainly based on Cr, Fe, Ca, and Ba concentrations.

MGO concentrations were 89–245% higher in site A compared to sites B, C, D, and E. The spatial variation of honey MGO concentrations aligns with inter- and intra-regional variation in *L. scoparium* nectar DHA previously observed by Williams et al. [19]. This is likely a result of genetic and environmental effects on nectar composition and yields [18,22]. Similarly, site A differed from other sites regarding the elemental composition of the honey. This is particularly true for Mn, which at site A was only 26–47% of the concentrations at sites B, C, D, and E. Furthermore, honey from site A had significantly lower Cu, K, Mg, Na, and S concentrations than sites B and C. Our findings are consistent with those of Grainger et al. [52], who showed that concentrations of Ca, K, Mg, Mn, and Na in honeys could be used to differentiate between the regions in New Zealand where the honeys were produced.

**Figure 3:**
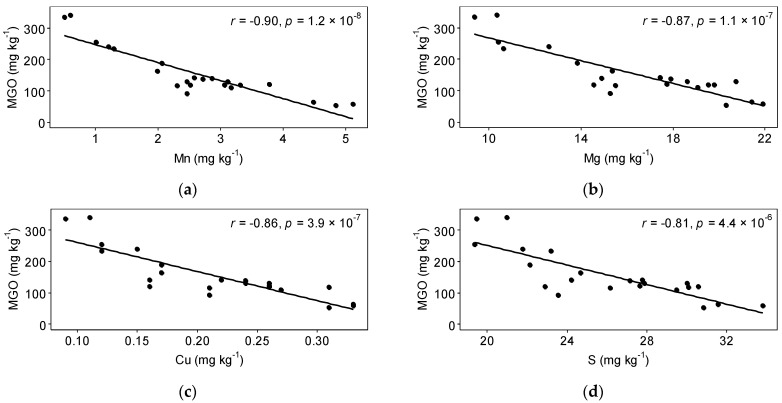
Honey MGO versus honey elemental concentrations: (**a**) Mn; (**b**) Mg; (**c**) Cu; (**d**) S. The black lines are linear regression lines. R values are Pearson’s correlation coefficients.

Negative correlations between honey elements and honey MGO were most pronounced for Mn, Cu, Mg, and S (Figure 3) but were also found for Na (*r* = −0.69, *p* ≤ 0.001), Ba (*r* = −0.61, *p* ≤ 0.01), K (*r* = −0.57, *p* ≤ 0.01), Zn (*r* = −0.57, *p* ≤ 0.01), and Al (*r* = −0.51, *p* ≤ 0.05). While there is no previous study correlating the concentration of mānuka honey elements and MGO, Alqarni et al. [43] studied the elemental composition of honeys in Saudi Arabia and included two New Zealand mānuka honeys with differing UMF. The UMF 18 honey in their study had lower concentrations of Mg, Mn, K, and Zn than the UMF 10 honey, but a higher Na concentration. They did not report Cu, S, Ba, and Al concentrations [43].

### 3.3. Interactions

**Figure 4:**
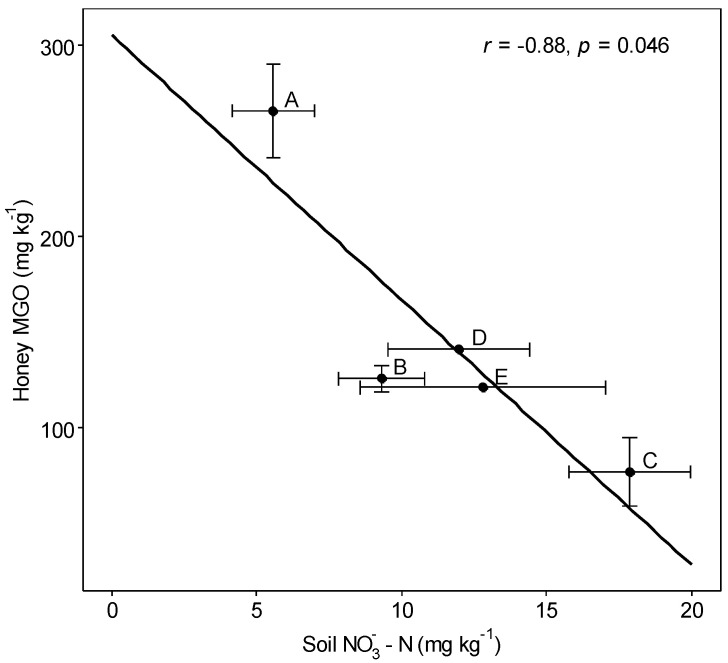
Honey MGO versus soil NO_3_^−^-N. Values are means for sites A-E (honey *n* = 1–9, soil *n* = 5–14). Bars represent the standard error of the mean. The black line is a linear regression line. R is the Pearson’s correlation coefficient.

Contrary to studies on other honeys [13,50], there were no correlations between the elemental composition of mānuka honey and elemental concentrations in soils. The only soil factor that correlated with honey MGO was soil NO_3_^−^ (*r* = −0.88, *p* < 0.05) (Figure 4). NO_3_^−^ was shown to accelerate *L. scoparium* root growth [37,38] and could increase the accumulation of Fe, Mn, Zn, and Cu [53]. However, soil NO_3_^−^ only correlated positively with plant Co (*r* = 0.49, *p* ≤ 0.005). It correlated negatively with plant Fe (*r* = −0.34, *p* ≤ 0.05). This could indicate a dilution of elements in the plants [40]. There was a positive correlation between the concentrations of K in plants and honeys (*r* = 0.91, *p* ≤ 0.01). Unlike most other elements tested, K is highly mobile in the plant phloem [54]. Therefore, elevated K concentrations in the plants may result in higher concentrations in the nectar.

The negative correlation between honey MGO and concentrations of Mn, Cu, Mg, S, Na, Ba, K, Zn, and Al does not necessarily indicate that these elements cause a reduction in honey antimicrobial activity. Both the honey MGO and elemental concentrations may correlate with another (unmeasured) factor. Direct effects of these elements in *L. scoparium* nectar on nectar DHA have not been described in the literature to date.

Smallfield et al. [55] found that DHA is not present in the phloem of *L. scoparium*, which indicates that its production is linked to nectar metabolism. Williams [26] suggested that DHA production might be associated with dihydroxyacetone phosphate production, which may occur in the floral nectaries [56]. The involved fructose 1, 6-bisphosphate requires Mg, Mn, Zn, or Co for activity [57]. In contrast, triosephosphate isomerase is inhibited by sulphate, phosphate, and arsenate [58]. Furthermore, in the nectar of *Nicotiana* spp., manganese superoxide dismutase generates high concentrations of H_2_O_2_ [59]. While H_2_O_2_ is only present at low levels in *L. scoparium* nectar [60], it can react with DHA to glycolate [61]. This indicates that, while the nectar and DHA production in *L. scoparium* is not understood, element-associated shifts in enzymatic reactions might affect levels of DHA in the nectar.

High concentrations of certain elements in nectar can also negatively affect the foraging behavior of honeybees. Xun et al. [62] showed that flowers treated with Zn, Cu, Ni, and Pb reduced the time honeybees spent foraging on these flowers and the amounts of nectar removal. Similarly, Meindl et al. [63] found that Ni-hyperaccumulation in plants reduced pollinator visitation. High concentrations of Mn and Cu in *L. scoparium* nectar might negatively affect the foraging behavior of honeybees and lead to more visitation of other nectar sources, which would therefore result in a lower MGO mānuka honey.

Previous studies have highlighted the importance of the botanical origin of honey as the main factor determining its elemental concentrations [64]. The lack of correlation between mānuka honey and *L. scoparium* foliage composition in this study indicates that honey elemental concentrations might have been diluted with other nectar. When honeybees gather nectar from different floral sources, the composition of the mānuka honey is affected. While mānuka honey has a higher total elemental concentration than non-native New Zealand honeys such as clover, native kāmahi (*Weinmannia racemose*) and rewarewa (*Knightia excelsa*) honeys have particularly high elemental concentrations [42]. High concentrations of chemical elements in this study could therefore be an indication of mānuka honey contamination with other floral nectar sources. *K. excelsa* is a common nectar contaminant that can result in dilution of mānuka honey [41]. Similarly, honeydew honey has high elemental concentrations [42]. *L. scoparium* is often infested by honeydew-producing scale insects, which leads to sooty mold development [65]. Sooty mold coverage was found to not directly affect the DHA concentration in *L. scoparium* nectar [19]. However, a dilution of mānuka honey with collected honeydew might affect the honey’s elemental composition. Furthermore, higher soil nutrient levels can be associated with increased exotic weed growth [66], which might in turn result in a dilution effect of mānuka honey with nectar from exotic species with high nectar elemental concentrations. It is therefore possible that the negative correlation between soil NO_3_^−^ and mānuka honey MGO in our study is a result of accelerated growth of other non-native vegetation.

It is also possible that the elements in our mānuka honey samples did not originate from floral nectar. Elements in honey can derive from environmental pollution, agrochemicals, or natural non-nectar sources that the bees are in contact with when foraging, including air, water, and soil [52,67,68]. Elements can also be introduced during honey processing [27]. Mānuka honey has a low pH of about 3.5–4.5 [69], which can result in contamination of honey with Zn from galvanized metal or Cr and Ni from stainless steel [70,71]. Furthermore, elemental concentrations in the honey may be changed when beekeepers use other sugar sources as bee feed [72].

While the chemical elements in mānuka honey may not be causative of honey quality, they may provide a low-cost indication of MGO levels and may be used as a quality indicator for honey. The lack of correlation between plant chemistry and honey MGO concentrations may be due to the large number of plants whence honeybees forage. Future work should test the hypothesis that higher concentrations of Mn, Cu, Mg, S, Na, Ba, K, Zn, and Al are associated with a lower DHA concentration in *L. scoparium* nectar.

## Figures and Tables

**Table 1:** Methylglyoxal (MGO), dihydroxyacetone (DHA), and elemental concentrations in honeys from sites A–E. The locations of the sites are shown in Figure 1.

Site	A	B	C	D	E
	*n* = 6	*n* = 9	*n* = 4	*n* = 1	*n* = 2
MGO	266 ± 25 ^a^	126 ± 6.9 ^b^	77 ± 18 ^b^	141	121
DHA	3246 ± 221 ^a^	1856 ± 71 ^b^	1293 ± 226 ^b^	1983	1642
Al	6.5 ± 0.91 ^a^	11 ± 0.60 ^b^	9.1 ± 0.34 ^ab^	5.1	5.5
B	2.8 ± 0.18 ^a^	2.8 ± 0.10 ^a^	2.5 ± 0.12 ^a^	2.8	2.8
Ba	0.08 ± 0.01 ^a^	0.09 ± 0.00 ^a^	0.11 ± 0.00 ^b^	0.09	0.09
Ca	61 ± 2.9 ^a^	60 ± 2.5 ^a^	61 ± 1.9 ^a^	62	68
Cr	0.02 ± 0.01 ^a^	0.02 ± 0.00 ^a^	0.02 ± 0.00 ^a^	0.02	0.03
Cu	0.13 ± 0.01 ^a^	0.24 ± 0.01 ^b^	0.30 ± 0.02 ^c^	0.16	0.21
Fe	1.2 ± 0.45 ^a^	1.1 ± 0.09 ^a^	0.91 ± 0.12 ^a^	0.72	0.95
K	487 ± 14 ^a^	671 ± 25 ^b^	1108 ± 55 ^c^	463	465
Mg	11 ± 0.68 ^a^	18 ± 0.61 ^b^	21 ± 0.35 ^c^	15	16
Mn	1.1 ± 0.23 ^a^	2.7 ± 0.15 ^b^	4.2 ± 0.60 ^c^	2.9	3.2
Na	27 ± 0.99 ^a^	34 ± 0.99 ^b^	47 ± 2.2 ^c^	38	40
P	54 ± 0.99 ^a^	50 ± 1.4 ^a^	65 ± 4.4 ^b^	62	64
S	21 ± 0.62 ^a^	28 ± 0.85 ^b^	31 ± 43 ^b^	24	25
Zn	0.32 ± 0.02 ^a^	0.36 ± 0.03 ^a^	0.47 ± 0.03 ^b^	0.37	0.47

Mean ± standard error. Different letters indicate significant differences between sites at *p* ≤ 0.05 according to Tukey’s HSD test. Values are in mg kg^−1^.

## Data Availability

The data presented in this study are available on request from the corresponding author.

## Supplementary Materials

The following are available online at https://www.mdpi.com/article/10.3390/foods10071670/s1, Table S1: Chemical characterization of soil at the different sites, Table S2: Soil exchangeable element concentrations at the different sites, Table S3: *L. scoparium* foliage elemental concentrations at the different sites.
