# Supplementary material for: Chemical Elements and the Quality of Mānuka (Leptospermum scoparium) Honey
Source: Foods. 2021 Jul 20;10(7):1670. doi: 10.3390/foods10071670 (PMC8303644; doi:10.3390/foods10071670)
Supplement: Supplementary file 1 [file foods-10-01670-s001.zip › foods-1281776-supplementary.pdf]

Chemical elements and the quality of mānuka (*Leptospermum scoparium*) honey

Supplementary material

Table S1: Chemical characterization of soil at sites A-E.

site	A		B		C		D		E	
n=	*10*		*14*		*5*		*5*		*5*	
NH_4^+-N	11	± 3.9^{a}	17	± 6.7^{a}	2.6	± 0.61^{a}	11	± 1.8^{a}	15	± 4.6^{a}
NO3$^-$-N	5.6	± 1.42^{a}	9.3	± 1.5^{ab}	18	± 2.1^{b}	12	± 2.5^{ab}	13	± 4.26^{ab}
pH	4.5	± 0.11^{ab}	4.5	± 0.09^{ab}	5.0	± 0.06^{b}	4.8	± 0.37^{ab}	4.2	± 0.05^{a}
N (%)	0.36	± 0.06^{a}	0.69	± 0.03^{b}	0.25	± 0.01^{a}	0.65	± 0.18^{b}	0.32	± 0.05^{a}
C (%)	4.9	± 0.95^{ab}	11	± 0.74^{c}	2.6	± 0.08^{b}	7.5	± 1.9^{ac}	5.2	± 0.57^{a}
C/N	13	± 0.33^{b}	16	± 0.5^{a}	11	± 0.33^{c}	12	± 0.35^{bc}	17	± 0.85^{a}
Al	31559	± 2339^{a}	51646	± 2115^{b}	30685	± 610^{a}	40267	± 3888^{ab}	36919	± 5195^{a}
As	3.6	± 0.66^{b}	4.0	± 0.17^{b}	4.2	± 0.23^{b}	4.0	± 0.55^{b}	2.1	± 0.26^{a}
B	27	± 0.7^{a}	35	± 1.3^{b}	47	± 1.0^{c}	42	± 5.0^{bc}	22	± 3.00^{a}
Ca	1854	± 337^{a}	1617	± 126^{a}	5171	± 75^{c}	4036	± 1193^{bc}	1848	± 135^{ab}
Cd	0.08	± 0.01^{a}	0.22	± 0.02^{b}	0.18	± 0.02^{bc}	0.11	± 0.02^{ac}	nd	
Cr	21	± 0.72^{a}	31	± 2.5^{b}	26	± 0.45^{ab}	28	± 2.2^{ab}	21	± 1.6^{a}
Cu	5.8	± 0.72^{a}	11	± 1.3^{b}	13	± 0.27^{b}	5.7	± 0.90^{a}	5.2	± 0.93^{a}
Fe	13064	± 316^{a}	19799	± 368^{b}	13249	± 60.7^{a}	14339	± 1138^{a}	12427	± 1086^{a}
K	3168	± 169^{a}	3339	± 314^{a}	8870	± 521^{b}	9057	± 1410^{b}	4860	± 161^{a}
Li	24	± 1.7^{ab}	36	± 1.9^{c}	35	± 0.28^{bc}	80	± 7.1^{d}	19	± 3.6^{a}
Mg	3910	± 250^{ab}	4699	± 200^{bc}	5499	± 43^{c}	4841	± 338^{bc}	3501	± 395^{a}
Mn	236	± 28^{a}	274	± 23^{a}	356	± 6.2^{a}	386	± 121^{a}	185	± 36^{a}
Na	276	± 17^{ab}	217	± 5.8^{a}	267	± 7.0^{ab}	318	± 54^{b}	224	± 20^{ab}
Ni	8.0	± 0.38^{a}	7.2	± 0.39^{a}	15	± 0.23^{b}	8.2	± 0.60^{a}	7.5	± 1.2^{a}
P	452	± 59^{b}	664	± 49^{c}	560	± 27^{bc}	878	± 140^{c}	152	± 16^{a}
Pb	12	± 0.52^{a}	19	± 0.45^{b}	22	± 1.2^{bc}	26	± 1.9^{c}	11	± 1.9^{a}
S	404	± 76^{a}	664	± 42^{c}	204	± 12^{b}	892	± 274^{c}	311	± 33^{ab}
Sr	19	± 1.3^{b}	24	± 1.5^{b}	24	± 0.72^{ab}	43	± 12^{a}	37	± 1.1^{a}
Zn	44	± 2.0^{a}	45	± 2.5^{a}	81	± 1.5^{b}	81	± 8.2^{b}	33	± 5.7^{a}

Mean ± standard error. Different letters indicate significant differences between sites ($p \leq 0.05$). Values are in mg kg^{-1} unless otherwise indicated.
nd=not detectable

Table S2: Soil exchangeable element concentrations at sites A-E.

site	A			B			C			D			E		
n=	*10*			*14*			*5*			*5*			*5*		
Al	121	±	18^{ab}	250	±	37^{a}	21	±	5.3^{c}	84	±	37^{bc}	154	±	16^{ab}
Cd	<0.01	±	0.00	<0.02	±	0.00	<0.01	±	0.00	nd			nd		
Co	0.19	±	0.05^{ac}	0.08	±	0.03^{b}	0.59	±	0.07^{c}	0.39	±	0.16^{ac}	0.13	±	0.04^{ab}
Cr	<0.02	±	0.00^{a}	0.02	±	0.00^{a}	0.00	±	0.00^{b}	0.02	±	0.01^{a}	0.01	±	0.00^{a}
Cu	<0.03	±	0.01^{ab}	0.02	±	0.00^{a}	0.05	±	0.01^{b}	0.02	±	0.00^{a}	0.01	±	0.00^{a}
Fe	28	±	8.6^{a}	96	±	23^{b}	2.6	±	0.46^{c}	14	±	9.8^{ac}	43	±	13^{ab}
Li	0.08	±	0.02^{ab}	0.06	±	0.01^{a}	0.18	±	0.01^{bc}	0.31	±	0.06^{c}	0.10	±	0.02^{abc}
Mg	223	±	60^{ab}	155	±	18^{a}	179	±	14^{ab}	460	±	162^{b}	262	±	44^{ab}
Mn	26	±	5.6^{a}	22	±	2.3^{a}	22	±	3.4^{a}	66	±	21^{a}	31	±	11^{a}
Na	73	±	12^{a}	59	±	4.4^{a}	26	±	3.0^{b}	105	±	27^{a}	96	±	19^{a}
Ni	0.19	±	0.04^{ab}	0.12	±	0.01^{a}	0.39	±	0.06^{b}	0.13	±	0.05^{a}	0.11	±	0.01^{ab}
P	2.9	±	1.1^{a}	2.4	±	0.22^{ab}	1.3	±	0.11^{a}	5.0	±	1.1^{b}	1.4	±	0.21^{a}
Zn	1.6	±	0.60^{a}	1.7	±	0.38^{a}	2.3	±	0.13^{a}	1.5	±	0.47^{b}	1.5	±	0.30^{a}

Mean ± standard error. Different letters indicate significant differences between sites ($p \leq 0.05$). Values are in mg kg^{-1}.

nd=not detectable

< actual mean is lower due to sample concentrations being below detection limit

Table S3: *L. scoparium* foliage elemental concentrations at sites A-E.

site	A			B			C			D			E		
n=	*10*			*15*			*5*			*5*			*5*		
N (%)	1.0	±	0.07^{a}	0.99	±	0.06^{a}	1.03	±	0.06^{a}	0.97	±	0.07^{a}	1.1	±	0.04^{a}
C (%)	49	±	0.31^{a}	49	±	0.24^{a}	50	±	0.39^{a}	49	±	0.33^{a}	49	±	0.30^{a}
C/N	49	±	3.4^{a}	52	±	2.9^{a}	49	±	2.4^{a}	52	±	4.2^{a}	46	±	2.1^{a}
Al	212	±	61^{a}	105	±	22^{a}	26	±	4.5^{b}	132	±	29^{a}	72	±	15^{ab}
As	<0.16	±	0.05^{a}	<0.24	±	0.04^{a}	<0.12	±	0.05^{a}	0.29	±	0.06^{a}	0.21	±	0.03^{a}
B	24	±	1.6^{a}	21	±	1.3^{a}	19	±	2.3^{a}	19	±	1.1^{a}	22	±	1.1^{a}
Ca	4610	±	466^{a}	4584	±	349^{a}	4102	±	365^{a}	5640	±	539^{a}	4548	±	524^{a}
Cd	<0.05	±	0.01^{a}	<0.06	±	0.02^{a}	<0.04	±	0.01^{a}	<0.07	±	0.02^{a}	<0.03	±	0.02^{a}
Co	<0.05	±	0.01^{a}	<0.01	±	0.00^{b}	0.08	±	0.02^{a}	<0.04	±	0.01^{ab}	0.12	±	0.05^{a}
Cr	4.5	±	2.2^{a}	0.63	±	0.08^{b}	0.43	±	0.16^{b}	1.1	±	0.28^{ab}	0.81	±	0.10^{ab}
Cu	4.1	±	0.50^{a}	3.9	±	0.32^{a}	3.3	±	0.42^{a}	2.7	±	0.25^{a}	4.6	±	0.60^{a}
Fe	214	±	57^{c}	84	±	16^{ab}	36	±	4.3^{a}	128	±	29^{bc}	66	±	12^{ab}
K	3887	±	168^{bc}	3958	±	124^{b}	5015	±	342^{d}	3080	±	159^{ac}	3000	±	289^{a}
Li	0.29	±	0.09^{a}	0.08	±	0.02^{b}	0.22	±	0.05^{a}	0.39	±	0.06^{a}	0.14	±	0.03^{ab}
Mg	1009	±	40^{c}	927	±	36^{c}	1094	±	106^{ac}	1468	±	102^{b}	1397	±	112^{ab}
Mn	322	±	53^{bc}	169	±	13^{b}	74	±	8.7^{d}	448	±	115^{ac}	874	±	165^{a}
Na	2435	±	212^{ab}	2054	±	154^{abc}	1634	±	153^{bc}	1415	±	163^{c}	2819	±	252^{a}
Ni	4.3	±	1.1^{a}	1.8	±	0.31^{a}	2.6	±	0.34^{a}	3.7	±	1.8^{a}	4.1	±	1.8^{a}
P	601	±	47^{ab}	561	±	30^{a}	633	±	48^{ab}	771	±	84^{b}	429	±	40^{a}
S	934	±	49^{b}	926	±	59^{b}	685	±	47^{b}	814	±	74^{b}	1254	±	103^{a}
Sr	24	±	3.0^{b}	35	±	2.5^{ab}	21	±	2.3^{b}	37	±	5.1^{ab}	43	±	9.8^{a}
Zn	21	±	1.8^{a}	23	±	1.7^{a}	17	±	1.9^{a}	26	±	5.5^{a}	25	±	2.1^{a}

Mean ± standard error. Different letters indicate significant differences between sites ($p \leq 0.05$). Values are in mg kg^{-1} unless otherwise indicated.

nd=not detectable

< actual mean is lower due to sample concentrations being below detection limit
